# A Case Report on Mycobacterium abscessus: An Emerging Pathogen

**DOI:** 10.7759/cureus.23072

**Published:** 2022-03-11

**Authors:** Asad Chohan, Saiara Choudhury, Pahnwat T Taweesedt, Rahul Dadhwal, Abhay P Vakil, Zuhair Ali, Rene Franco

**Affiliations:** 1 Department of Pulmonology, Corpus Christi Medical Center Bay Area, Corpus Christi, USA

**Keywords:** disseminated, pulmonary and extrapulmonary disease, ntm, nontuberculous mycobacteria, mycobacterium abscessus

## Abstract

The incidence of infections by rapidly growing mycobacteria has increased in recent decades. nontuberculous mycobacteria(NTM) *represent over 190 species and subspecies and can cause both pulmonary and extrapulmonary symptoms. *The* Mycobacterium abscessus *complex (MABC)is among the most drug-resistant mycobacterial species, and prompt diagnosis and effective eradication are burdensome. We present the clinical course of a 55-year-old female who was diagnosed with *M. abscessus *and explore her clinical diagnosis and possible treatment options. This case report emphasizes the challenges clinicians face in the prompt diagnosis of *M. abscessus* and discusses the treatment options in light of the recent guidelines.

## Introduction

Over 190 species and subspecies of nontuberculous mycobacteria (NTM) have been documented, many of which are known to produce disease in humans. They can affect both pulmonary and extrapulmonary sites [1]. Over the past few decades, there has been a notable increase in the number of NTM cases. *Mycobacterium abscessus* is the most common and rapidly growing NTM and is among the most drug-resistant pathogens, hence accounting for limited therapeutic options and a high treatment failure rate [2]. Although there is a consensus that the incidence and prevalence of NTM cases have been on the rise globally, they are not well-documented or reported like tuberculosis, and an accurate assessment of incidence remains difficult. Prompt diagnosis based on clinical suspicion has been a challenge, but an even major obstacle is the complete eradication of the disease. *M. abscessus* can cause skin and soft tissue infections after trauma or surgical procedures, and pulmonary infections and disseminated diseases in immunocompromised patients.

## Case presentation

**Figure 1:**
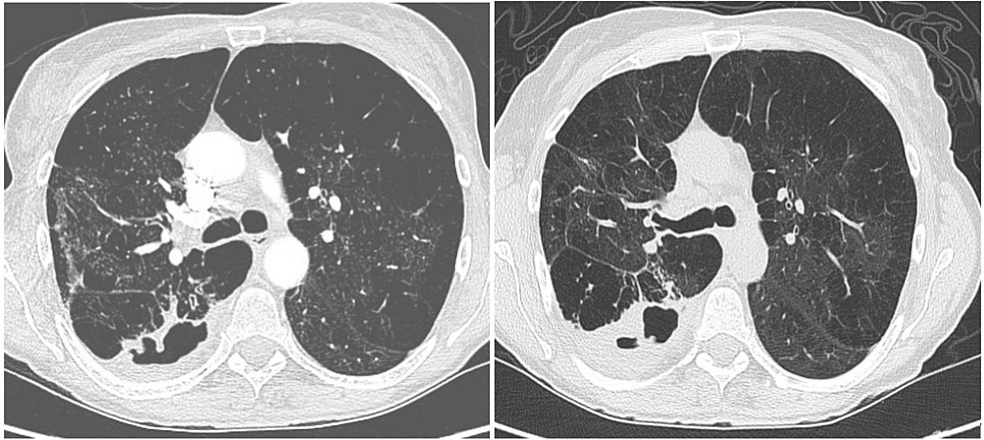
Computed tomography scan of the chest at the level of tracheal bifurcation showed cavitary lesion at the first visit (left) and second visit (right)

A 55-year-old White female with hypertension, chronic obstructive pulmonary disease (not on home oxygen), and a chronic cough presented to the emergency room (ER) with a worsening cough, low-grade fever, multiple episodes of small amounts of hemoptysis, and subjective weight loss over the past three months. She was an active smoker and reported that her symptoms had worsened for the past week before she arrived at the ER. The shortness of breath and cough (which was productive with white sputum) were also accompanied by diffuse, bandlike chest pain. The patient denied any significant weight loss, hemoptysis, or night sweats. The patient had a history of bronchoscopy performed nine months ago for bronchiectasis seen on the CT scan chest (Figure 1A), and her bronchoalveolar lavage (BAL) culture was positive for *M. abscessus* complex (MABC). She was then started on a four-month course of antibiotics and scheduled to follow up with an infectious disease (ID) clinic. The patient initiated her treatment regimen with improved symptoms but did not complete the full course of antibiotic treatment and was lost to follow-up until this visit. The patient states that her recent symptoms started within two months of stopping her antibiotic therapy.

On physical examination, the patient appeared cachectic but was hemodynamically stable without respiratory failure. A high-resolution computed tomography (HRCT) scan of the chest was done, which revealed a cavitary lesion (Figure 1B.). Sputum cultures were sent, which eventually came back positive for "Mycobacterium abscessus." After discussion with the patient, a course of three antibiotic regimens was initiated.

## Discussion

The *M. abscessus* complex is a complex of rapidly-growing mycobacteria (RGM) species of NTM which is second in prevalence after the *M. avium* complex (MAC). MABC often causes severe respiratory, skin, and mucosal infections in humans. This species is also considered the most antibiotic-resistant of all the mycobacteria [3]. As per a study, it was estimated that MABC represented 3-13% of all NTM-PD [4]. Out of the MABC, *M. abscessus* is the most common isolate, followed by *M. massiliense* and *M. bolletii* [2]. However, the proportion of the three subspecies may vary according to geographical distribution [5]. In terms of diagnosis, high suspicion is required for the diagnosis of NTM in general and MABC in particular, as clinically and radiographically it closely mimics other chronic or subacute infections, malignancies, and autoimmune pathologies. Moreover, in parts of the world with a high prevalence of tuberculosis, NTM diagnosis is often missed and treatment of tuberculosis can be started, which has an even worse impact on NTM resistance. A study demonstrated that a large percentage of diagnosed cases of TB in Brazil were NTM cases, and patients received the wrong set of medications with many potential side effects. In high burden areas of TB, NTM-PD has often been mistaken for multidrug-resistant tuberculosis (MDR-TB) [6,7]. Isolating a sputum sample and culturing for MABC can also be ineffective due to a high sputum contamination rate in certain settings. Generally, bronchoscopy with BAL is needed, with a sample sent to a specialized lab for diagnosis. However, it is at this step where the real challenge in identifying MABC and subsequent treatment based on antibiotic sensitivity lies.

Different techniques are used in the detection of NTMs and the classification of them accordingly. As per the initial classification in the 1970s, there was no classification past the *M. abscessus-chelonae* group. However, with time, the difference between MABC and *M. chelonae* in terms of genotype as well as pathogenicity and antibiotic resistance has become more and more clear. Therefore, there is a dire need to evolve the classification system of MABC based on pathogenetic characteristics. With the possibility of complete genetic sequencing, a consensus list of the targeted genes for classification needs to be adopted and followed by most diagnostic labs. Currently, there are no FDA-approved commercial DNA probes that completely sequence MABC species based on the 16StRNA gene. Furthermore, the ability to detect the erm41 gene to further classify MABC into subspecies is also essential to determine macrolide sensitivity [8].

Antibiotic resistance is also the biggest challenge in the treatment and eradication of all NTM infections, but most notably in MABC infections. It is important to never empirically start an NTM infection on antibiotic therapy as it can worsen the resistance and the empiric antibiotic therapy may not be appropriate. Appropriate sensitivities are always necessary in MABC cases. Macrolides remain the cornerstone for most NTM therapies, and the species sensitive to macrolides have higher treatment-response and clearance rates. The erm41 gene is responsible for macrolide resistance in two of the three subspecies of MABC, namely *M. abscessus* and *M. bolletii*. *M. massiliense* subspecies is generally macrolide sensitive with better outcomes. Generally, in-vitro macrolide resistance may not be detected, but in-vivo Macrolide resistance is always present in the presence of the mentioned gene [9]. Another cornerstone of antibiotic therapy in MABC infections is Amikacin, and resistance to Amikacin also indicates a high possibility of failure of disease remission. There are cases of resistance to Amikacin too, and the treatment-failure or re-infection rate is quite high in MABC infection.

Classically, for NTM treatment, long-term directed antibiotic therapy is given with serial monitoring for drug side effects and repeat sampling to confirm disease eradication. As mentioned, this strategy is often ineffective in MABC cases. An alternative strategy for the management of the disease can also be adopted. In asymptomatic diseases or those with minimal symptoms and low life expectancy, avoiding antibiotic therapy can be a feasible solution in certain cases. In other cases where the disease is quite localized with a particular lung area being affected, thoracotomy can be of benefit as long as the patient can tolerate the surgery. Another target for antibiotic therapy could be symptomatic relief, as severe symptoms can be eradicated by antibiotic therapy even without achieving disease eradication [9].

In our case, despite high suspicion and diagnosis of NTM in general, appropriate classification into MABC with macrolide sensitivity was not performed. Based on the lab report, the "*M. abscessus chelonae* complex" was identified. Empiric treatment with an antibiotic regimen was proposed, while surgical evaluation was also started. However, due to advanced COPD and deconditioning, plans for surgery were postponed. With empiric antibiotic therapy, improved symptoms were nevertheless observed.

## Conclusions

Our case aims to highlight "*M. abscessus chelonae*" as a fast-growing, pathogenic nontuberculous mycobacterium species with significant challenges in diagnosis and management, thereby requiring a robust multidisciplinary approach. Long-term targeted antibiotic therapy, despite significant side effects, can prove inadequate in order to permanently clear the infection. However, it usually halts progression and provides symptomatic relief. The worsening structural disease often results in patients who are partially treated. Our case also throws light on the need for close outpatient follow-up and long-term management strategies.
